# Medical News and Miscellany

**Published:** 1891-06

**Authors:** 


### Medical News and Miscellany.

Died, at Green Springs, Ohio, May 8th, 1891, Dr. Wm. Ball, of Castroville, Texas.

Mrs. Dr. T. G. Richardson has given $100,000 to Tulane University, to build a new Medical Department.

Dr. R. C. Hodges, of Galveston, the oculist, has just returned from New York, where he took a special course in eye surgery at the Polyclinic.

Dr. J. C. Culbertson, the game cock editor of the Cincinnati *Lancet and Clinic*, has been made editor and business manager of the Journal of the American Medical Association.

**Surgeon-General Swearingen.**—It is not generally known, that, under the laws of Texas the State Health Officer is ex-officio Surgeon-General of the State, and chief of the Governor's medical staff. Such is the fact.

---

**Dr. J. B. Hamilton** has resigned the office of Supervising Surgeon General, and accepted a chair in the Medical College, Chicago. Well, well. We thought he had gotten the M. H. S. fixed to suit him, and was fixed lor lifie.

---

**Dr. I. N. Love**, the genial editor of the *Medical Mirror*, after a three months illness, and absence for rest and recuperation, is back at his post; and having donned the editorial robes once more, we shall look to see some unusually brilliant reflections in that *Mirror*. Success to it and its gifted editor.

---

**Dr. Matt. M. Smith**, who was a pupil of Dr. W. A. Morris, of Austin, graduated at Jefferson Medical College last session, having been chosen President of the *Alumni* of 1890–1. This is a high compliment to be bestowed on a student, and is a credit to Texas. By the bye, it is getting quite common for Texas students to carry off the honors and prizes. Dr. Smith has located at Austin.

---

**Two Excellent Journals for the Price of One.**—DANIELS' TEXAS MEDICAL JOURNAL, $2, and the *Kansas City Medical Index*, $1. To all new subscribers to Vol. 7, and all old subscribers who pay up arrears and renew, paying for Vol. 7 in advance, we will send both journals for one year for $2! In other words, we will give the *Kansas City Medical Index as a premium* to cash subscribers remitting the subscription price of the JOURNAL a year in advance.

---

**Exhibition of the First Case of Lupus Treated by Koch's Lymph.**—H. Levy, of Berlin, at a meeting of the German Surgical Society (April 1 to 4, 1891), exhibited the first case of lupus treated by Koch's lymph. The treatment had continued six months. Patient not well yet. Some parts of the disease had cicatrized, others had not been affected at all. New deposits had appeared on the foot and fingers, where there had been no desease.—*Deutsche Med. Zeitung.*—*The Med. Bulletin.*

**Removals.**—Dr. W. B. Anderson has removed from Llano to Content, Runnels county.

Dr. T. P. Roberts has removed from Jasper to Colmesneil.

Dr. J. W. Miller has removed from Itaska to Hillsboro.

Dr. E. R. Walker has removed from Schulenburg to Halletsville.

Dr. J. W. Harris has removed from Dripping Springs to Brownwood.

---

**"Straws."**—The Antikamnia Chemical Co., of St. Louis, write: "We have had very appreciable business results from our advertisement in your JOURNAL." (Only four months in.—ED.) Sharp & Dohme renew their contract this month, for the *seventh consecutive year.*

Dr. R. W. Hayden, President of the New York Pharmacal Co., of Bedford Springs, Massachusetts, manufacturers of the famous "Hayden's Viburnum Compound,' renews for the fourth consecutive year, prepaying the entire year's contract.

Dr. R. W. Gardner, the New York Manufacturing Chemist, renews for the third consecutive year. The Missouri Medical College renews for the sixth consecutive year, and enlarges their ad. to half page.

If all this does not indicate a healthy tone, and show that advertising in DANIEL'S TEXAS MEDICAL JOURNAL pays the advertiser, what would do so? Send for rates.

---

**Through an oversight** upon my part, the name of Dr. S. S. McCrum, of Emory, Texas, was omitted in the list of graduates of the medical department of the University of Nashville and Vanderbilt University, as published in the announcement of these schools for the session of 1891-92. Such omission is calculated to do a most worthy gentleman and accomplished physician great injustice, hence this card.

W. L. NICHOL, M. D.,
Registrar, Med. Dept. Univ. of Nashville and Vandbt. Univ.

---

**The College of Physicians and Surgeons** of New York held its annual commencement on the 10th, inst. The graduating class numbered 173, of whom 53, or 30 per cent. we rejected, 121 receiving the coveted diploma. This is an unusually large per cent. of failures. It indicates a very healthy tone, and shows that he who would win distinction in that college must work. The ten young men whose examination was the most thorough and satisfactory constituted the honor roll, and the JOURNAL is much gratified to see on the list, away up, near "head," the name of Seth Morris, of Austin. He is a son and student of Dr. W. A. Morris, of this city. We learn that Dr. Morris will locate in Austin.

---

**Official Report on Koch's Remedy.**—The German governmedt has obtained statistics from fifty-nine hospitals, where, during eight weeks, 2172 patients were treated with 17,500 injections of the lymph. Of 1061 patients, suffering from internal tuberculosis, 13 were cured, 171 much improved, 194 slightly improved, 587 no better, and 46 died. Out 708 sufferers from external tuberculosis 15 were cured, 148 much improved, 237 slightly improved, 298 no better, and 9 died. Owing to the reported failnres the "Tuberculin" is in little demand from the Berlin chemists, while at Madrid the committee appointed to examine the treatment, have checked any further experiments. The only success reported from Spain is stated to have been with lepers and persons suffering from lupus.—London letter in *Journal A. M. A., May 23.*

Referring to the above figures we have 1769 patients suffering from tuberculosis and 28 cures in two months. This gives a little more than one and a half per cent. cures. In Loomis' latest edition on Practice of Medicine, page 225, he says, "Recovery has occurred in one-sixth of my recorded cases during the past ten years." This remark refers to the treatment of chronic pulmonary tuberculosis by all the means at his command, viz.: by drugs, hygiene, climate, etc., and shows sixteen and two-thirds per cent. recoveries. Flint's Practice, 5th edition, 1881, p. 211, gives eleven per cent. recoveries from the best treatment,—drugs, hygienic, climatic, etc., and over five and sixteenth per cent. recoveries without any treatment at all. Thus it is seen that Koch's treatment is over four per cent. less successful than no treatment at all.

